# Consumption of fruits and vegetables and its association with sleep duration among Finnish adult population: a nationwide cross-sectional study

**DOI:** 10.3389/fnut.2024.1319821

**Published:** 2024-05-16

**Authors:** Anupa Thapa, Tuuli Lahti, Mirkka Maukonen, Timo Partonen

**Affiliations:** ^1^Doctoral School of Health Sciences, University of Helsinki, Helsinki, Finland; ^2^Finnish Institute for Health and Welfare, Helsinki, Finland; ^3^Master School, Health and Wellbeing, Turku University of Applied Sciences, Turku, Finland

**Keywords:** fruits and vegetable consumption, sleep duration, chronotype, dietary habits, public health nutrition

## Abstract

**Introduction:**

Sleep and diet are crucial determinants of overall health and wellbeing, with the potential to mutually influence each other. This study examined the association between sleep duration and fruits and vegetables (FV) consumption among Finnish adults.

**Methods:**

The study analyzed data from the National FinHealth 2017 Study involving 5,043 adults aged 18 years and above. Participants reported their habitual sleep duration, and dietary consumption through a validated self-administered questionnaire. Confounders such as demographic, socio-economic factors, and chronotype were considered. A sensitivity analysis, which excluded energy under-reporters, was conducted to validate the findings.

**Results:**

Mean dietary consumption was compared across three sleep duration categories (short, normal, long), revealing that short sleepers consumed 37 g/d fewer FV, and long sleepers consumed 73 g/d fewer FV than normal sleepers. Binary logistic regression analyses consistently demonstrated significant negative association between FV consumption and both short and long sleep duration across all models, even when adjusted for a range of covariates. Linear regression analyses revealed a positive but non-significant association between sleep duration and FV consumption that became significant when excluding energy under-reporters, particularly in model 1.

**Discussion:**

This study suggests a consistent pattern where deviation from normal sleep duration was associated with decreased FV consumption, suggesting the need for considering sleep patterns in dietary intervention. The substantial role of accurate energy reporting in explaining these associations is highlighted. Further research, including longitudinal studies, is needed to better understand the mechanisms underlying these associations.

## Introduction

Sleep and diet are critical determinants of overall health and wellbeing (1). It is recommended that a healthy adult should sleep between 7 to 9 h a night (2, 3). However, the modern landscape, shaped by lifestyle dynamics and environmental triggers such as sedentary habits and dietary choices, has led to a concerning surge in adult sleep deprivation (4, 5). Finland, for instance, has witnessed an upswing in sporadic insomnia symptoms (6) accompanied by reduced sleep duration in adult population (7). This emerging public health concern is amplified by the link between inadequate sleep and adverse health outcomes, including cardiovascular diseases (8), cognitive malfunction (9), and increased all-cause mortality (10).

Recognizing the intricate relationship between health outcomes and dietary choices (11), substantial efforts have been directed toward enhancing people’s food-related knowledge (12–14). However, certain aspects of modern life, such as time constraints, easily accessible fast foods, stress, and inadequate sleep, collectively contribute to unhealthy food choices (15, 16). The World Health Organization (WHO) recommends a daily consumption of at least 400 g of fruits and vegetables, a guideline underpinned by the well-established benefits of substantial FV intake in mitigating the risk of chronic diseases (17–19). The recently updated Nordic Nutrition Recommendations for 2023 advocate an even higher daily consumption of at least 500 to 800 g of vegetables, fruits, and berries, with half of the consumption coming from vegetables (20). Unfortunately, data from many countries, including Finland shows non-compliance (21–24). In Finland, for example, only 14% of men and 22% of women met the current national recommendation of consuming a minimum of 500 g of vegetables, fruits, and berries per day (25, 26).

While the significance of adequate sleep and consumption of FV for overall health is becoming more evident through numerous studies, a notable gap exists in comprehending the potential interplay between these factors within the general population. Existing research has primarily concentrated on specific demographic segments, including pregnant women, adolescents, children, young adults, and the elderly, often with limited sample sizes (27–33). Consequently, a comprehensive understanding of these associations across a broader spectrum of the population remains elusive. To bridge this gap, our study embarked on a nationwide cross-sectional analysis encompassing a larger and more diverse adult cohort.

Chronotype, which reflects an individual’s preference for timing of activities during the day (morning or evening preference), has demonstrated its influence on both sleep patterns and dietary choices in prior research (34–37). However, previous studies investigating the link between sleep duration and FV consumption have often overlooked chronotype as a potential confounder. Including chronotype in the analysis allows us to delve into how an individual’s innate preference for activity timing interacts with their sleep duration and dietary behaviors. This aspect of our study is pivotal as it contributes an additional layer of understanding when examining the association between sleep duration and dietary behaviors.

Our study provides a holistic perspective on the interactions between sleep duration and fruits and vegetable consumption. We aim to specifically explore how sleep duration influence fruits and vegetable consumption and *vice-versa*. Additionally, we will investigate the role of chronotype as a potential confounder in the associations between sleep duration and fruits and vegetables consumption.

## Methods

The data for this study were derived from the National FinHealth 2017 Study, which is a population-based cross-sectional health survey. It comprises of a representative sample of individuals aged 18 and older residing in Finland. Participants were selected through stratified random sampling methods based on gender, age, and geographical location. Out of the eligible sample (*n* = 10,247) who received an invitation letter to a health examination and to complete a self-administered questionnaire by mail, 58.1% participated in the health examination. During this examination, participants were provided with additional questionnaires, including the Food Frequency Questionnaire (FFQ), which could also be completed electronically. Of those who took part in the health examination 86.1% returned the FFQ. Exclusions from the study were made for various reasons: incomplete FFQs (110 cases), duplicate responses (9 cases) and withdrawal of consent (7 cases). To ensure the credibility of the dietary data, FFQs that reported extreme and implausible energy intake values were also excluded (51 FFQs). This was based on daily energy intake thresholds that marked the lowest and highest 0.5% of sex-specific energy intake for monitoring dietary intake in large cohort studies (38, 39). After these exclusions, the total sample size was narrowed to 5,125 participants. A further exclusion was made due to missing information on sleep duration (80 cases), resulting in a final analytical sample of 5,043 adults for this study. The pattern of missing data was evaluated using Little’s MCAR test, which yielded a Chi-Square statistic of 2.167 with one degree of freedom (*p* = 0.14), suggesting that the missingness was random. As a result, listwise deletion was employed to handle missing data in the analysis.

The study protocol and method of the FinHealth 2017 study is described in detail elsewhere (40, 41). Ethical approval for the FinHealth 2017 Study was obtained from the Coordinating Ethics Committee for the Hospital District of Helsinki and Uusimaa (Reference 37/13/03/00/2016), and all participants provided written informed consent. In addition, for this study a permission was acquired from the institutional review board at the Finnish Institute for Health and Welfare (THL) to further analyze the dataset (Reference FT2019_025). All procedures were performed in accordance with the valid guidelines and regulations as well as the Declaration of Helsinki and its amendments.

### Study variables

This study examined two pivotal variables: sleep duration and total fruit and vegetable consumption (TFVC). Sleep duration was assessed through a self-administered questionnaire. Participants were asked to report their habitual sleep duration with the question, “How many hours do you sleep in 24 h?” They were instructed to provide their response in hours and minutes, reflecting their average sleep pattern. For subsequent analysis, minutes were converted into hours, and the total sleep duration was then computed. The resulting total sleep duration was further classified into three categories: short sleepers (less than 7 h per day), normal sleepers (7–9 h per day, the reference group), and long sleepers (more than 9 h per day).

The primary dietary variable of interest was the consumption of fruits (including citrus fruits, apple, berries, other fresh and canned fruits) and vegetables (including green leafy vegetables, root-vegetables, cabbages, mushrooms, legumes, fruit vegetables, other fresh and canned vegetables). The dietary information was gathered using a validated 134-item self-administered semi-quantitative food frequency questionnaire (FFQ) (42–44). Participants reported their habitual diet over the past 12 months, indicating the average consumption of each food item with a scale of 10 frequency categories ranging from none to six or more times a day (none, less than once a month, 1–3 times a month, once a week, 2–4 times a week, 5–6 times a week, once a day, 2–3 times a day, 4–5 times a day, and 6 or more times a day). The portion sizes were predefined and expressed in common household and natural units (e.g., glass, or slice). The average daily food consumption (g/day) and energy intake were calculated using the FINESSI software of THL and the Finnish National Food Composition Database (FINELI) (39).

### Background variables

The FinHealth 2017 Study included information on background variables including gender, age, education, employment, household income, cohabitation, the number of household members, the number of living children, Body Mass Index (BMI), physical activity level, smoking, alcohol consumption, total energy intake and chronotype. These variables were included in the statistical regression models as control variables, reflecting their established associations with sleep duration in previous literature.

For further analysis, age, education, employment, household income, cohabitation, body-mass index (BMI), physical activity level and chronotypes were grouped into different categories. Participants were categorized by age into younger adults (18–34 years), middle-aged adults (35–64 years), or older adults (65 years or older). BMI was grouped in four categories as follows: BMI < 18.5 (underweight), BMI 18.5–24.9 (normal weight), BMI 25.0–29.9 (overweight), or BMI > 30.0 (obese).

Physical activity levels over the previous 12 months were assessed by four categories: inactive (light activities such as reading and watching television); moderately active (walking, gardening or other activities ≥ 4 h/week); active (running, swimming or other physically demanding activities ≥ 3 h/week); or very active (competition or other heavy sports several times/week). The categories “active” and “very active” were combined for the present study as only a few participants were classified as “very active.”

Chronotype was assessed using self-evaluation questions with four available options: “definitely a morning type,” “rather more a morning than an evening type,” “rather more an evening than a morning type” and “definitely an evening type.” Participant’s response was further categorized into morning type, intermediate type (combining “rather more a morning than an evening type” and “rather more an evening than a morning type,” and evening type during analysis).

Education levels were expressed as basic (elementary/basic/lower secondary education), intermediate (vocational/upper secondary/high school/non-university lower education), or higher (college/university education). Working status was categorized as employed (paid job/self-employed/unpaid employment in a family-owned business, apprenticeship, and paid internship), or outside of work (unemployed/student/unpaid internship/retired/on family leave/stay-at-home mother/father).

Household income was grouped based on annual income before tax reduction as low (lowest through 35,000 €), middle (35,001 to 60,000 €), or high (60,001 € and higher). Participants in married/cohabitated/registered partnership were grouped as living together, whereas those who were single/separated/divorced/widowed were grouped as living alone.

### Statistical analyses

Descriptive statistics were calculated, and FV consumption compared between sleep categories using analyses of covariance, followed by the Bonferroni multiple comparisons post-hoc test with adjustments for age, gender and energy intake were made where relevant.

Linear regression analysis was employed to explore the potential influence of sleep duration on FV consumption, employing three models with increasing levels of covariate adjustment. Model 1 adjusted for basic covariates such as age, gender and total energy intake. Model 2 further adjusted for an expanded set of covariates, including BMI, education, employment, marital status, household income, the number of household members, the number of living children, smoking, and alcohol intake. Model 3 included all the covariates from Model 2, along with chronotype as an additional covariate.

Binary logistic regression analysis was undertaken to explore the potential influence of FV consumption across different sleep categories: short sleep vs. normal sleep, and long sleep vs. normal sleep, adjusting for the same set of covariates used in the linear regression analysis. To estimate the magnitude of differences between normal sleepers and short or long sleepers, effect sizes were calculated using Cohen’s d and the effect-size correlation rYλ.

A sensitivity analysis was conducted by repeating both the linear and logistic regression analyses after excluding participants identified as energy under-reporters. Energy misreporting was assessed by calculating the ratio of reported energy intake to predicted Basal Metabolic Rate (BMR). Participants with an energy intake to BMR ratio (EI:BMR) of 1.14 or less were considered under-reporters and were excluded from this secondary analysis (39).

All statistical analyses were performed using the IBM Statistical Package for Social Sciences (SPSS) Statistics, Version 28 (International Business Machines Corporation, Armonk, NY, United States).

## Results

Table 1 depicts the distribution of the study population across different sleep duration categories. Of the 5,043 participants, 21% were short sleepers, 76.1% were normal sleepers, and the remaining 2.9% were long sleepers. The mean sleep duration for short sleepers was 6.0 h (SD = 0.6), for normal sleepers 7.7 h (SD = 0.6), and for long sleepers 10.1 h (SD = 0.7). The majority of the participants identified themselves as intermediate types (61.7%), while 22.4% identified as morning types and 15.9% as evening types. The mean age of the participants was 55 years (SD = 16.0), with 55.9% being female, and 71.2% being in marital, cohabiting, or registered relationships. The education levels showed diversity: 19.2% held basic education, 49.3% intermediate, and 31.5% higher education. Regarding employment, half of the participants were employed, and the other half were not part of the workforce. The participants’ income levels varied, with 36.4% falling within the low-income bracket, 32.0% within middle income, and 312.6% within higher income bracket. Household configurations displayed variety, with 71.6% having 1–2 members, 26.8% having 3–5 members, and 1.7% exceeding 5 members. In terms of children in the household, 22.5% had none, 69.2% had 1–3 children, and 8.3% had more than 3 children. Regarding substance use, 17.1% smoked daily, and 20.9% consumed alcohol at a risk level. Notably, nearly half of the participants were overweight (40.4%) or obese (25.1%). Physical activity levels varied, with 46.9% of participants reporting a moderately active level followed by 29.0% who were active and 24.1% who were inactive.

**Table 1:** Study population by sleep categories.

Characteristics	Sleep duration			
	Short sleepers (<7 h/day)	Normal sleepers (7–9 h/day)	Long sleepers (>9 h/day)	Total
Observations (*n*)	1,063 (21.0%)	3,831 (76.1%)	149 (2.9%)	5,043 (100%)
Mean sleep duration (h) *(SD)*	6.0 *(0.6)*	7.7 *(0.6)*	10.1 *(0.7)*	7.4 *(1.0)*
(95%CI)	(5.9–6.0)	(7.6–7.7)	(10.0–10.3)	(7.3–7.4)
Chronotype	*n = 4,937*			
Morning type	296 (6.0%)	784 (15.9%)	26 (0.5%)	1,106 (22.4%)
Intermediate type	561 (11.4%)	2,403 (48.7%)	81 (1.6%)	3,045 (61.7%)
Evening type	176 (3.6%)	572 (11.6%)	38 (0.8%)	786 (15.9%)
Age (years)	*n* = 5,043			
18–34	107 (2.1%)	594 (11.8%)	12 (0.2%)	713 (14.1%)
35–64	597 (11.8%)	2,076 (41.2%)	62 (1.2%)	2,735 (54.2%)
≥65	359 (6.8%)	1,161 (22.5%)	75 (1.5%)	1,595 (31.6%)
Mean age, years *(SD)*	57 *(15.1)*	54 *(16.1)*	62 *(16.9)*	55 *(16.0)*
(95% CI)	(56–57)	(53–54)	(59–64)	(54–55)
Gender	*n* = 5,043			
Male	499 (9.9%)	1,665 (33.0%)	59 (1.2%)	2,223 (44.1%)
Female	564 (11.2%)	2,166 (43.0%)	90 (1.8%)	2,820 (55.9%)
Cohabitation	*n* = 5,036			
Living together	695 (13.8%)	2,786 (55.3%)	106 (2.1%)	3,587 (71.2%)
Living alone	364 (7.2%)	1,042 (20.7%)	43 (0.9%)	1,449(28.8%)
Education	*n* = 5,032			
Basic level	258 (5.1%)	671 (13.3%)	37 (0.7%)	966 (19.2%)
Intermediate level	534 (10.6%)	1,870 (37.2%)	77 (1.5%)	2,481 (49.3%)
Higher level	268 (5.4%)	1,283 (25.5%)	34 (0.7%)	1,585 (31.5%)
Employment	*n* = 5,034			
Employed	532 (10.6%)	1,959 (38.9%)	26 (0.5%)	2,517 (50.0%)
Outside workforce	531 (10.5%)	1,863 (37.0%)	123 (2.4%)	2,517 (50.0%)
Household annual income	*n* = 4,901			
Low income (≤35,000€/a)	390 (8.0%)	1,314 (26.8%)	81 (1.7%)	1,785 (36.4%)
Middle income (35,001–60,000€/a)	330 (6.7%)	1,196 (24.4%)	42 (0.9%)	1,568 (32.0%)
Higher income (≥60,000€/a)	306 (6.2%)	1,221 (25.9%)	21 (0.4%)	1,548 (31.6%)
Household members (n)	*n* = 5,022			
1–2 members	778 (15.5%)	2,688 (53.5%)	128 (2.5%)	3,594 (71.6%)
3–5 members	263 (5.2%)	1,063 (21.2%)	18 (0.4%)	1,344 (26.8%)
>5 members	17 (0.3%)	64 (1.3%)	3 (0.1%)	84 (1.7%)
Children in the household (*n*)	*n* = 4,956			
No children	189 (3.8%)	889 (17.9%)	35 (0.7%)	1,113 (22.5%)
1–3 children	748 (15.1%)	2,587(52.2%)	96 (1.9%)	3,431 (69.2%)
>3 children	113 (2.3%)	282 (5.7%)	17 (0.3%)	412 (8.3%)
Currently smoking	*n* = 5,043			
Yes	194 (3.8%)	642 (12.7%)	28 (0.6%)	864 (17.1%)
No	869 (17.2%)	3,189 (63.2%)	121 (2.4%)	4,179 (82.9%)
Alcohol risk use	*n* = 5,007			
No	835 (16.7%)	3,014 (60.2%)	112 (2.2%)	3,961 (79.1%)
Yes	218 (4.4%)	794 (15.9%)	34 (0.7%)	1,046 (20.9%)
BMI (kg/m^2^)	*n* = 4,979			
Underweight	10 (0.2%)	23 (0.5%)	0 (0%)	33 (0.7%)
Healthy weight	297 (6.0%)	1,352 (27.2%)	37 (0.7%)	1,686 (33.9%)
Overweight	437 (8.8%)	1,516 (30.4%)	58 (1.2%)	2,011 (40.4%)
Obese	301 (6.0%)	896 (18.0%)	52 (1.0%)	1,249 (25.1%)
Mean BMI, kg/m^2^ *(SD)*	28.0 (5.3)	27.2 *(4.8)*	28.7 *(5.9)*	27.4 *(5.0)*
(95% CI)	(27.7–28.3)	(27.0–27.3)	(27.7–29.6)	(27.2–27.5)
Physical activity level	*n* = 4,999			
Inactive	318 (6.4%)	823 (16.5%)	65 (1.3%)	1,206 (24.1%)
Moderately active	472 (9.4%)	1,815 (36.3%)	56 (1.1%)	2,343 (46.9%)
Active	261 (5.2%)	1,161 (23.2%)	28 (0.6%)	1,450 (29.0%)

Table 2 displays the mean differences in the consumption of various FV across different sleep duration categories. It was observed that normal sleepers had higher consumption of FV including all sub-groups when compared to both short and long sleepers. These differences were statistically significant after adjusting for covariates including age, gender, and energy intake with significance maintained at the 0.05 level following Bonferroni correction for multiple comparisons.

**Table 2:** Mean difference in consumption of fruits and vegetables across sleep duration categories.

	Normal sleepers (7–9 h/day)	Short sleepers (<7 h/day)		Long sleepers (>9 h/day)	
	Mean consumption g/day (95% CI)	Mean difference^*^ (95% CI)	*p-*values^**^	Mean difference* (95% CI)	*P*-values^**^
Total fruits and vegetables	463.1 *(455.6 to 470.6)*	−37.0 *(−56.7 to −17.4)*	<0.001	−73.4 *(−120.6 to −26.1)*	<0.001
Total vegetables	285.0 *(279.7 to 290.3)*	−23.0 *(−36.8 to −9.2)*	<0.001	−42.5 *(−75.8 to −9.1)*	0.007
Green leafy vegetables	19.1 *(18.6 to 19.6)*	−1.5 *(−2.8 to −0.1)*	0.023	−4.4*(−7.7 to −1.2)*	0.003
Root vegetables	41.0 *(39.9 to 42.2)*	−3.5 *(−6.5 to −0.5)*	0.014	−1.4 *(−8.6 to 5.8)*	1.00
Fruit vegetables	154.9 *(151.2 to 158.9)*	−14.9 *(−24.4 to −5.4)*	<0.001	−32.0 *(−54.8 to −9.1)*	0.002
Other fresh and canned vegetables	70.2 *(68.5 to 71.9)*	−3.1 *(−7.5 to 1.4)*	0.29	−4.6 (−15.3 to 6.1)	0.91
Total fruits	178.2 *(174.2 to 182.3)*	−14.2 *(−24.8 to −3.5)*	0.004	−31.0 *(−56.7 to −5.3)*	0.013
Citrus fruits	30.8 *(29.3 to 32.3)*	−1.2 *(−5.1 to 2.7)*	1.00	−0.08 *(−9.4 to 9.6)*	1.00
Apple	56.3 *(54.3 to 58.3)*	−4.4 *(−9.7 to 0.8)*	0.13	−18.9 *(−31.6 to −6.1)*	0.001
Berries	34.8 *(33.7 to 35.9)*	−4.1 *(−6.9 to −1.2)*	0.002	−5.4 *(−12.2 to 1.4)*	0.18
Other fresh and canned fruits	56.8 *(55.2 to 58.4)*	−4.5 *(−8.7 to −0.3)*	0.031	−6.4 *(−16.6 to 3.8)*	0.41

*P*-values significant at the 0.05 level. CI, Confidence interval.^*^The mean difference is significant at 0.05 level. The model was adjusted for covariates (age, gender, and energy intake).^**^Adjustment for multiple comparisons: Bonferroni.

Effect sizes measured by Cohen’s d, were small but notable. For normal vs. short sleepers, Cohen’s d was 0.16 and for normal vs. long sleepers, it was 0.21. Corresponding effect-size correlations were 0.08 and 0.10, respectively, suggesting a small yet positive association between sleep duration and FVs consumption.

In the vegetable sub-group, significant differences were observed in the consumption of green leafy vegetables, root vegetables and fruit vegetables (e.g., tomatoes, cucumbers), between normal and short sleepers. Similarly, for normal vs. long sleepers, significant differences were again noted for green leafy vegetables and fruit vegetables. However, other fresh and canned vegetables such as cabbage, mushroom, onion, peas and beans did not exhibit significant differences.

In the fruit sub-groups, a significant mean difference was observed in the consumption of berries and other fresh and canned fruits between normal and short sleepers. Conversely, for normal vs. long sleepers, the only significant difference was observed in apple consumption.

Table 3 presents the results of linear regression analyses exploring the association between sleep duration and TFVC, along with its sub-groups. Initially, the association between sleep duration and TFVC was observed to be positive across all models, though it did not reach statistical significance. This trend persisted even with the inclusion of additional covariates with slight decrease in the strength of the association in Models 2 and 3, which did not significantly impact the associations.

**Table 3:** Association between sleep duration and FV consumption.

Variables	Model 1		Model 2				Model 3		
Sleep duration	B (95% CI)	*P*-value	Adjusted R^2^	B (95% CI)	*P*-value	Adjusted R^2^	B (95% CI)	*P*-value	Adjusted R^2^
TFVC	6.2 (−0.1 to 12.5)	0.055	0.24	4.8 (−1.6 to 11.3)	0.14	0.29	4.7 (−1.8 to 11.2)	0.16	0.29
*Green leafy vegetables*	0.1 (−0.3 to 0.6)	0.48	0.09	0.3 (−0.2 to 0.7)	0.26	0.12	0.17 (−0.3 to 0.6)	0.46	0.12
*Root vegetables*	0.9 (−0.1 to 1.8)	0.07	0.10	0.7 (−0.3 to 1.7)	0.16	0.10	0.80 (−0.2 to 1.8)	0.12	0.11
*Fruit vegetables*	1.8 (−1.2 to 4.9)	0.24	0.12	1.6 (−1.5 to 4.8)	0.31	0.15	1.2 (−2.0 to 4.4)	0.47	0.15
Other fresh and canned vegetables	0.9 (−0.5 to 2.3)	0.21	0.13	0.9 (−0.6 to 2.4)	0.24	0.14	0.7 (−0.7 to 2.2)	0.32	0.14
*Citrus fruits*	0.7 (−0.5 to 2.0)	0.25	0.05	0.6 (−0.7 to 1.9)	0.37	0.06	0.7 (−0.6 to 2.1)	0.28	0.06
*Apple*	0 (−1.6 to 1.7)	0.96	0.07	−0.6 (−2.3 to 1.2)	0.53	0.09	−0.4 (−2.2 to 1.4)	0.65	0.09
*Berries*	0.8 (−0.1 to 1.7)	0.08	0.16	0.4 (−0.5 to 1.4)	0.38	0.20	0.5 (−0.4 to 1.4)	0.30	0.20
Other fresh and canned fruits	0.9 (−0.4 to 2.3)	0.17	0.10	0.9 (−0.5 to 2.3)	0.20	0.12	0.9 (−0.5 to 2.4)	0.20	0.12

Covariates for model 1: age, gender, and total energy intake. Covariates for model 2: age, gender, total energy intake, BMI, education, employment, marital status, household income, the number of household members, the number of living children, smoking, alcohol intake and physical activity level. Covariates for model 3: age, gender, total energy intake, BMI, education, employment, marital status, household income, the number of household members, the number of living children, smoking, alcohol intake, physical activity level and chronotype. *P*-values significant at the 0.05 level. CI, Confidence interval.

However, when a sensitivity analysis was conducted by excluding energy under reporters (*n* = 1,926)-the association between sleep duration and TFVC in Model 1 became statistically significant (B = 11.9, 95% CI [2.6, 21.3], *p* = 0.012), indicating a stronger relationship after removing potential bias from underreporting. Although this significance was nearly reached in Models 2 and 3 (*p* = 0.06), the proximity to significance suggests that energy underreporting might be attenuating the true magnitude of the observed relationships.

In the subgroup analysis, the pattern was somewhat similar, with certain food groups such as root vegetables and other fresh and canned vegetables showing a significant increase in the strength of association in the sensitivity analysis compared to the full cohort. For instance, root vegetables showed a significant association with sleep duration in Model 1 after excluding underreporters (B = 1.6, *p* = 0.029). The association for other fresh and canned vegetables also became significant (B = 2.6, *p* = 0.015 in Model 1) in the sensitivity analysis.

Conversely, no significant associations were observed for green leafy vegetables, fruit vegetables, citrus fruits, apples, berries, and other fresh and canned fruits after excluding energy underreporters, similar to the original analysis.

Table 4 presents the outcomes of binary logistic regression models that examined how the consumption of various fruits and vegetables is associated with the likelihood of short or long sleep durations, using normal sleep as the reference category.

**Table 4:** Association between FV consumption and sleep duration categories.

	Model 1		Model 2				Model 3		
Major food groups	Beta	Wald	*P*-value	Beta	Wald	*P*-value	Beta	Wald	*P*-value
**Short sleep vs. normal sleep**									
Total fruits and vegetables	−0.001	21.5	<0.001	−0.001	13.8	<0.001	−0.001	13.6	<0.001
*Green leafy vegetables*	−0.006	6.9	0.005	−0.006	5.6	0.018	−0.005	4.6	0.033
*Root vegetables*	−0.003	8.3	0.004	−0.003	8.2	0.004	−0.004	9.1	0.003
*Fruit vegetables*	−0.001	14.0	<0.001	−0.001	9.6	0.002	−0.001	8.6	0.003
Other fresh and canned vegetables	−0.001	2.8	0.09	−0.001	2.7	0.098	−0.001	2.5	0.11
*Citrus fruits*	−0.001	0.6	0.44	0	0.1	0.73	0	0.1	0.69
*Apple*	−0.001	4.0	0.045	−0.001	1.6	0.21	−0.001	1.7	0.19
*Berries*	−0.004	11.7	<0.001	−0.003	6.8	0.009	−0.003	5.9	0.015
Other fresh and canned fruits	−0.002	6.7	0.010	−0.002	3.8	0.049	−0.002	4.8	0.029
**Long sleep vs. normal sleep**									
Total fruits and vegetables	−0.001	12.9	<0.001	−0.001	6.5	0.011	−0.001	6.4	0.012
*Green leafy vegetables*	−0.021	9.7	0.002	−0.013	3.6	0.059	−0.015	4.8	0.028
*Root vegetables*	−0.001	0.3	0.59	−0.001	0.3	0.56	−0.001	0.2	0.64
*Fruit vegetables*	−0.003	10.2	0.001	−0.002	5.0	0.026	−0.002	5.5	0.019
Other fresh and canned vegetables	−0.002	1.1	0.30	−0.002	1.2	0.27	−0.002	1.0	0.31
*Citrus fruits*	0	0	0.9	0	0	0.83	0.001	0.2	0.69
*Apple*	−0.006	11.3	<0.001	−0.005	7.5	0.006	−0.005	6.6	0.010
*Berries*	−0.005	3.3	0.06	−0.003	1.5	0.22	−0.002	0.6	0.42
Other fresh and canned fruits	−0.003	2.0	0.15	−0.001	0.4	0.51	−0.002	0.7	0.40

Covariates for model 1: age, gender, and total energy intake. Covariates for model 2: age, gender, total energy intake, BMI, education, employment, marital status, household income, the number of household members, the number of living children, smoking, alcohol intake and physical activity. Covariates for model 3: age, gender, total energy intake, chronotype, BMI, education, employment, marital status, household income, the number of household members, the number of living children, smoking, alcohol intake, physical activity and chronotype. *P*-values significant at the 0.05 level.

For individuals with short sleep duration, a consistent negative association with TFVC was observed across all models. This pattern suggests that short sleepers tend to consume fewer FV compared to those with normal sleep duration (*p* < 0.001). A similar negative trend was observed for specific sub-groups such as green leafy vegetables, root vegetables, fruit vegetables, and berries, though the strength and significance of association varied across the models. Conversely, no significant associations were found for the consumption of other fresh and canned vegetables and citrus fruits (*p* > 0.05), except for apple consumption, which showed a negative association with short sleep only in Model 1.

Among long sleepers, similar negative associations were reported with TFVC that were significant in Models 1 and 3. The consumption of green leafy vegetables showed a notable reduction in model 1, and TFVC showed a consistent negative association across all models. However, no significant associations were found for root vegetables, other fresh and canned vegetables, and citrus fruits in any models. The relationship with apple consumption remained significantly negative across all models for long sleepers, while the associations for berries and other fresh and canned fruits did not maintain significance in Models 2 and 3.

Unlike linear regression, in the sensitivity analysis, the logistic regression revealed a persistent negative association between short sleep duration and total fruit and vegetable consumption (Beta = −0.001, *p* < 0.001 across all models), indicating that the findings were stable even when potentially biased self-reported energy intake was accounted for. Similarly, the negative associations for specific sub-groups such as green leafy vegetables, root vegetables, and fruit vegetables remained significant, with minor variations in Beta coefficients and Wald statistics. For long sleepers, the sensitivity analysis generally showed a slight decrease in the strength of associations, yet the negative trend in total fruit and vegetable consumption remained significant in Models 1 and 3, with similar patterns observed for specific sub-groups. This sensitivity analysis result confirms the validity of our original findings, suggesting that the associations between TFVC and sleep duration are not substantially altered when controlling for energy underreporting. The detailed outcomes of the sensitivity analysis are presented in Supplementary Table 2.

Overall, the results indicate that both short and long sleep durations were associated with a decrease in the consumption of certain fruits and vegetables when compared to normal sleep durations.

## Discussion

This study examined the associations between FV consumption and sleep duration in a population-based cohort of Finnish adults, revealing a significant association between sleep duration and FV consumption. This included various sub-groups such as green leaf, root and fruit vegetables. Our findings are in line with existing literature, which consistently demonstrates reduced FV consumption among individuals with either inadequate or excessive sleep durations (27, 29, 30, 32, 33, 45). A closely related study by Noorwali et al. (31), focused on similar exposure and outcome variables in UK adults aged 19–65. Both studies categorized sleep duration into short, normal, and long sleepers, enabling direct comparisons. However, distinctions in dietary assessment methods exist. Noorwali et al. using a 4-day food diary validated with biomarkers, while our study employed a 12-month FFQ, assessing habitual diet and providing insights into long-term dietary patterns. Despite these methodological differences, both studies consistently reported reduced FV consumption among individuals with inadequate or excessive sleep durations.

One possible explanation for the observed negative association between short and long sleepers and FV consumption, as supported by previous studies, is that individuals with normal sleep durations are more likely to adopt healthy lifestyles. These lifestyles are often characterized by high FV consumption, regular physical activity, and better quality of life (46). Hormonal mechanisms might also play a role. Certain FVs such as cherries, kiwi, tomatoes, and cucumbers contain high levels of melatonin, a hormone vital for regulating circadian rhythms and sleep patterns (47). The significant associations we observed between sleep duration and specific FV sub-groups, such as root, fruit, and green leafy vegetables, further support these notions.

Moreover, fruits and vegetables serve as abundant sources of a variety of micronutrients and non-nutrient bioactive compounds, including vitamins, phytochemicals like (poly) phenolic compounds and carotenoids, vital minerals such as potassium, calcium, and magnesium, and dietary fiber. Their impact on human health is significant, attributed to their medicinal properties such as anti-inflammatory, antimicrobial, antioxidant, anticancer, and their preventive effects against various chronic diseases (48). It is plausible to suggest that the broader spectrum of health-promoting properties inherent in various fruits and vegetables, as outlined by their bioactive components, may have influenced the observed associations between sleep duration and specific FV-subgroups.

In our analysis, we observed that including a comprehensive set of covariates, did not significantly change the observed association between sleep duration and FV consumption. This suggests that the relationship between sleep duration and FV consumption is robust and remains consistent across different models. In our study, we expanded beyond the conventional covariates, such as age, gender, BMI, physical activity level and socioeconomic status, by incorporating chronotype as a covariate in Model 3. This addition did not significantly alter the associations, indicating that these dietary behaviors are associated with sleep duration independently of the chronotype. The inclusion of chronotype as a covariate was informed by emerging research indicating its influence on dietary patterns. Studies have shown that evening chronotypes are often associated with unhealthy dietary behaviors, including a propensity for obesity-related eating habits (34, 49, 50). However, our findings suggest that while chronotype may play a role in general dietary preferences and behaviors, its impact on the specific relationship between sleep duration and FV consumption is minimal. This is evident from the consistency of the association between sleep duration and various types of FV consumption, as indicated by the similar B values and *p*-values observed across Models 2 and 3 in both linear and logistic regression analyses.

Furthermore, we examined the likelihood of individuals falling into specific sleep duration categories based on their dietary habits, providing insights into the predictive value of sleep patterns for FV consumption. Our finding highlights that sleep duration categories can serve as predictive factors for FV consumption, albeit with relatively small effect sizes. A similar finding was observed in another study, which reported that shorter sleep duration at night was associated with lower FV consumption the following day (33). This reinforces the idea that sleep patterns may have relevance in understanding dietary choices, as well as in formulating dietary interventions. Additional research including longitudinal studies is essential to understand these complex relationship and to ascertain its implications on public health and lifestyle intervention.

### Strengths and limitations

A notable strength of our study is the utilization of a large, randomly selected population-based sample, which enhances the statistical power and potential generalizability of our findings. Nonetheless, it is important to acknowledge certain limitations. One key aspect is our approach to managing missing data. We employed listwise deletion based on the assumption, supported by Little’s MCAR test (Chi-Square = 1.400, df = 2, *p* = 0.497) that the data was missing completely at random (MCAR). However, the potential for non-random missingness cannot be entirely ruled out. While listwise deletion is a valid approach under the MCAR condition, the potential for non-random missingness remains, and if the missing data are not MCAR, it could introduce bias and affect the generalizability of our results.

Furthermore, the reliance on self-reported data for sleep duration and food consumption may introduce recall and reporting biases such as under-or over-reporting. To address this issue, our statistical analyses included adjustments for factors like age, gender, and total energy intake. These adjustments, particularly for energy intake, help to some extent in mitigating biases related to dietary reporting. However, it is important to note that such adjustments cannot fully eliminate the inherent limitations of self-reported dietary data. Additionally, the Finnish Food Frequency Questionnaire (FFQ), though widely validated and ensuring comprehensive coverage, presents inherent limitations. Its length, comprising 134-items, may result in participant fatigue and consequent reporting errors influenced by participant characteristics.

These limitations, combined with the cross-sectional nature of our study, imply that we can identify associations, but causality cannot be inferred.

## Conclusion

In summary, this study highlights a significant link between sleep duration and FV consumption among adults. The associations persisted across most FV subgroups, even after conducting sensitivity analysis, suggesting a strong and consistent relationship. While the findings indicate modest effect sizes, they carry substantial statistical and practical implications. Targeted interventions focusing on FV sub-groups with pronounced associations, such as green leafy vegetables and fruit vegetables can lead to impactful behavior change. Additional research, particularly longitudinal studies, is needed to better understand these associations and their public health implications, especially in regions with similar population structures and dietary patterns to Finland.

## Data availability statement

The data analyzed in this study is subject to the following licenses/restrictions: this study used data from National FinHealth Study conducted by THL Finland. Data can be made available from THL upon reasonable request. Requests to access these datasets should be directed to admin.biobank@thl.fi.

## Ethics statement

The studies involving humans were approved by THL Ethics Committee eettinentoimikunta@thl.fi. The studies were conducted in accordance with the local legislation and institutional requirements. The participants provided their written informed consent to participate in this study.

## Author contributions

AT: Conceptualization, Data curation, Formal analysis, Investigation, Methodology, Software, Visualization, Writing – original draft, Writing – review & editing. TL: Conceptualization, Supervision, Writing – review & editing. MM: Methodology, Validation, Writing – review & editing. TP: Conceptualization, Methodology, Resources, Supervision, Writing – review & editing.
