# Supplementary material for: Consumption of fruits and vegetables and its association with sleep duration among Finnish adult population: a nationwide cross-sectional study
Source: Front Nutr. 2024 May 16;11:1319821. doi: 10.3389/fnut.2024.1319821 (PMC11137275; doi:10.3389/fnut.2024.1319821)
Supplement: Supplementary file 1 [file Table_1.docx]

| **Variables** | **Model 1** | | | **Model 2** | | | | **Model 3** | | |
| --- | --- | --- | --- | --- | --- | --- | --- | --- | --- | --- |
| **Sleep duration** | B (95% CI) | P-value | Adjusted  R^2^ | | B (95% CI) | P-value | Adjusted  R^2^ | B (95% CI) | P-value | Adjusted R^2^ |
| **TFVC** | 11.9 (2.6 to 21.3) | 0.012 | 0.17 | | 8.9 (-0.4 to 18.3) | 0.06 | 0.22 | 9.1 (-0.4 to 18.5) | 0.06 | 0.22 |
| Green leafy vegetables | 0.4 (-0.2 to 1.1) | 0.16 | 0.05 | | 0.6 (0 to 1.3) | 0.059 | 0.09 | 0.5 (-0.1 to 1.2) | 0.12 | 0.08 |
| Root vegetables | 1.6 (0.2 to 3.0) | 0.029 | 0.07 | | 1.2 (-0.2 to 2.7) | 0.10 | 0.09 | 1.4 (-0.1 to 2.9) | 0.07 | 0.09 |
| Fruit vegetables | 4.1 (-0.4 to 8.5) | 0.07 | 0.08 | | 3.9 (-0.7 to 8.4) | 0.09 | 0.11 | 3.3 (-1.3 to 8.0) | 0.15 | 0.11 |
| Other fresh and canned vegetables | 2.6 (0.5 to 4.7) | 0.015 | 0.11 | | 2.3 (0.1 to 4.5) | 0.036 | 0.12 | 2.1 (0 to 4.3) | 0.052 | 0.12 |
| Citrus fruits | 0.2 (-1.7 to 2.1) | 0.82 | 0.03 | | -0.1 (-2.1 to 1.9) | 0.90 | 0.04 | 0.1 (-1.9 to 2.1) | 0.93 | 0.04 |
| Apple | 0.4 (-2.0 to 2.9) | 0.74 | 0.04 | | - 0.8 (-3.3 to 1.8) | 0.55 | 0.08 | -0.5 (-3.1 to 2.1) | 0.70 | 0.08 |
| Berries | 1.2 (-0.1 to 2.6) | 0.08 | 0.12 | | 0.7 (-0.7 to 2.1) | 0.32 | 0.16 | 0.8 (-0.6 to 2.2) | 0.28 | 0.16 |
| Other fresh and canned fruits | 1.0 (1.0 to 3.1) | 0.31 | 0.06 | | 0.8 (-1.3 to 2.9) | 0.45 | 0.09 | 0.9 (-1.2 to 3.1) | 0.38 | 0.09 |

**Supplementary Table 1. Association between FV consumption and sleep duration (excluding energy under-reporters).**

*Covariates for model 1: age, gender, and total energy intake.*

*Covariates for model 2: age, gender, total energy intake, BMI, education, employment, marital status, household income, the number of household members, the number of living children, smoking, alcohol intake and physical activity level.*

*Covariates for model 3: age, gender, total energy intake, BMI, education, employment, marital status, household income, the number of household members, the number of living children, smoking, alcohol intake, physical activity level and chronotype. CI = Confidence interval, p-value significant at level 0.05.*

**Supplementary Table 2. Association between FV consumption and sleep duration categories (excluding energy under-reporters).**

|  | **Model 1** | | | **Model 2** | | | | | **Model 3** | | |
| --- | --- | --- | --- | --- | --- | --- | --- | --- | --- | --- | --- |
| **Major food groups** | Beta | Wald | P-value | | Beta | Wald | P-value | Beta | | Wald | P-value |
| **Short sleep vs. normal sleep** |  |  |  | |  |  |  |  | |  |  |
| Total fruits and vegetables | -0.001 | 16.1 | <0.001 | | -0.001 | 12.6 | <0.001 | -0.001 | | 13.3 | <0.001 |
| Green leafy vegetables | -0.007 | 7.0 | 0.008 | | -0.008 | 7.7 | 0.006 | -0.008 | | 7.1 | 0.008 |
| Root vegetables | -0.004 | 7.2 | 0.007 | | -0.004 | 7.5 | 0.006 | -0.004 | | 8.3 | 0.004 |
| Fruit vegetables | -0.001 | 12.8 | <0.001 | | -0.001 | 10.6 | 0.001 | -0.001 | | 10.2 | 0.001 |
| Other fresh and canned vegetables | -0.002 | 4.3 | 0.038 | | -0.002 | 4.4 | 0.035 | -0.002 | | 4.2 | 0.040 |
| Citrus fruits | 0 | 0.1 | 0.78 | | 0 | 0 | 0.97 | 0 | | 0 | 0.86 |
| Apple | -0.001 | 2.3 | 0.13 | | -0.001 | 0.7 | 0.41 | -0.001 | | 1.1 | 0.30 |
| Berries | -0.003 | 4.4 | 0.035 | | -0.002 | 2.3 | 0.13 | -0.002 | | 1.9 | 0.17 |
| Other fresh and canned fruits | -0.002 | 3.8 | 0.051 | | -0.002 | 2.9 | 0.09 | -0.002 | | 3.9 | 0.047 |
| **Long sleep vs. normal sleep** |  |  |  | |  |  |  |  | |  |  |
| Total fruits and vegetables | -0.001 | 9.1 | 0.003 | | -0.001 | 6.4 | 0.012 | -0.001 | | 6.4 | 0.011 |
| Green leafy vegetables | -0.019 | 6.0 | 0.014 | | -0.014 | 3.4 | 0.06 | -0.016 | | 4.3 | 0.039 |
| Root vegetables | -0.002 | 0.5 | 0.46 | | -0.002 | 0.6 | 0.44 | -0.002 | | 0.5 | 0.49 |
| Fruit vegetables | -0.003 | 6.6 | 0.010 | | -0.002 | 4.6 | 0.031 | -0.002 | | 4.8 | 0.028 |
| Other fresh and canned vegetables | -0.002 | 1.1 | 0.29 | | -0.003 | 1.6 | 0.21 | -0.003 | | 1.6 | 0.20 |
| Citrus fruits | -0.001 | 0.1 | 0.81 | | -0.001 | 0.1 | 0.80 | 0 | | 0 | 0.93 |
| Apple | -0.007 | 9.9 | 0.002 | | -0.007 | 7.5 | 0.006 | -0.007 | | 7.2 | 0.007 |
| Berries | -0.003 | 1.1 | 0.29 | | -0.002 | 0.6 | 0.45 | -0.002 | | 0.2 | 0.61 |
| Other fresh and canned fruits | -0.002 | 1.3 | 0.25 | | -0.001 | 0.3 | 0.026 | -0.002 | | 0.5 | 0.46 |

*Covariates for model 1: age, gender, and total energy intake.*

*Covariates for model 2: age, gender, total energy intake, BMI, education, employment, marital status, household income, the number of household members, the number of living children, smoking, alcohol intake and physical activity level.*

*Covariates for model 3: age, gender, total energy intake, BMI, education, employment, marital status, household income, the number of household members, the number of living children, smoking, alcohol intake, physical activity level and chronotype. CI = Confidence interval, p-value significant at level 0.05..*
